# The ISRCTN Register: achievements and challenges 8 years on

**DOI:** 10.1111/j.1756-5391.2011.01138.x

**Published:** 2011-08

**Authors:** Helene Faure, Iain Hrynaszkiewicz

**Affiliations:** ^1^Editorial Director, Databases Current Controlled TrialsUK; ^2^Journal Publisher, BioMed CentralUK

## Abstract

The ISRCTN register has been operational for the past 8 years and is approaching 10,000 trial records. It complies with international guidelines and pools its data in the International Trial Search Portal initiated by the World Health Organisation. Through its ongoing collaboration with the Department of Health in England, the register has been able to participate in a national initiative aiming to bring clinical trials to the attention of a wider public with the objective of maximising participation. As part of BioMed Central, the register provides the first step in the concept of threaded publications, enabling the tracking of clinical research studies from inception and the linking of all resulting publications including the raw data where this is available.

## Introduction

The ISRCTN Register administered by Current Controlled Trials (CCT) has been in existence for over 8 years and is one of the established public trial registers vetted by the International Committee of Medical Journal Editors (ICMJE) and pooled in the World Health Organization (WHO) trials search platform (1).

The register has always enjoyed a close collaboration with UK-based organisations including the Department of Health in England. However, its remit remains international and it is open to all areas of healthcare and all study designs in all countries, for trialists who wish to declare the existence of their research, in line with international recommendations and at the request of a growing number of scientific journals.

## Achievements & trends

In response to the growing body of opinion in favor of prospective registration of randomized trials, the Science Navigation Group launched the CCT website in 1998. The aim was to facilitate the exchange of information about randomized trial worldwide and to publish an increasing number of trials protocols and trial results via its publishing business, BioMed Central (2).

CCT initially offered the metaRegister of Controlled Trials (mRCT), a platform bringing together different sources of trial information, allowing those providers to customize the presentation of their research and helping users to search those sources in a single environment. In 2003, acknowledging the fact that the same trial could be present in several registers pooled in the metaRegister (for example a register representing a funding body overlapping with another register representing a disease area), CCT started the ISRCTN (International Randomized Controlled Trial Number) scheme to allocate a unique trial ID to a trial against a basic set of trial information. The aim was for the trial ID to be quoted on all resulting publications.

In 2006, the PubMed LinkOut system was enabled to recognize trial IDs. When they are quoted in the abstract of an article, those trials IDs are stored in the PubMed secondary ID field and a banner of the trial register (where all the trial information is held) is displayed at the top of the record. This allows users to go to the original ISRCTN trial registration details (3).

Over the years, the ISRCTN dataset has been expanded in line with international guidelines to cater for the needs of the scientific community and the general public. It also includes information on ethics approval, trial end date, patient information sheet, trial website, as well as the WHO minimum data set (4). The register also seeks to keep abreast of future requirements, such as the inclusion of basic trial results. In considering the trends over time discussed below, it is important to keep in mind the caveat that public registration (defined as voluntary declaration of the existence of a piece of research, to comply with guidelines from the research community or funding bodies) is not compulsory. As a result, an increase or decrease of registration will reflect the amount of focus put on the matter, the funding made available for public registration, or both. It cannot be used to indicate that there have been more or fewer trials at any particular time.

Within the UK, the ISRCTN register is not the result of national legislation and is not supported by grants or governmental budgets. From 2003, an administrative fee has been charged for assigning ISRCTNs and hosting trial records on a website that users can access free and without login. This is in line with the open access journal publishing model developed by CCT's sister company, the Open Access publisher BioMed Central, where authors pay an Article Processing Charge which covers the cost of the publication of their research article, so that the research can be made freely available to all.

The requirement for trial registration in publicly available registers gained momentum in 2005 with declarations from the ICMJE (5). To ensure compliance with international guidelines, ISRCTN registration activities were also handed over to a not-for-profit organization ISRCTN.org.

The ISRCTN register proactively contacts trialists to update their data, especially regarding publications, and also processes any requests for updates that come from trialists. ISRCTN records are never removed from the ISRCTN, ensuring that research that is not published is still listed, reducing publication bias and making sure those trials are not missed by systematic reviewers (6).

As of June 2011, the ISRCTN Register has the following features:

- Over 9700 records (7) (Tables 1 and 2)
- From over 90% in the early years, the UK now contributes fewer than 55% of all the trials registered. The register has always been positioned as open to registrations from all countries. The proportion of non-UK trials has increased as the existence of the ISRCTN register has become more widely known internationally and the register has been offering a reliable registration service for trialists who are based in countries which may not yet have a local register or who need to comply with international regulations and journals’ recommendations. Countries with an increased research output such as China, Brazil and India are represented in the top 20 countries (Table 3) but it has to be noted that these countries have also been developing their own trial registers and more and more registers are becoming data providers to the WHO search portal (8).
- Medical journals very much focus on trials of interventions and that is reflected in the low number of observational trials listed (Table 4). The register was initially launched to list randomized trials but is now open to all study designs. Over 95% of the content still deals with interventional studies and randomized trials.
- In line with ICMJE guidelines, the aim is to make sure that trials are publicly registered prospectively, i.e. before the first participants are enrolled and randomized. For the ISRCTN register, a trial is registered retrospectively if the application is made before the anticipated start date of the trial (minus a grace period of 30 days). In practice, the majority of trials are still registered retrospectively, although the percentage of prospective registration has increased significantly over the years (Table 5). The ISRCTN register accepts retrospective registration, whether the trial is ongoing or completed at time of registration, because an inclusion in a database such as the ISRCTN register is still considered a better option than no inclusion at all. Furthermore, ICMJE member journals do not yet fully adhere to the ICMJE registration policy, and allow retrospective registration to take place (9).

**Table 1:** Growth in the number of trials in the ISRCTN

Year	Number of trials added in year	Cumulative total	Annual increase
2000	145	145	-
2001	187	332	129%
2002	302	634	91%
2003	729	1363	115%
2004	1065	2428	78%
2005	1493	3921	61%
2006	1304	5225	33%
2007	1220	6445	23%
2008	908	7353	14%
2009	836	8189	11%
2010	1146	9335	14%

**Table 2:** Proportion of trials in ISRCTN that are from the UK

	Total ISRCTNs added	UK related trials	% of UK trials in ISRCTN database
2000	145	133	92%
2001	187	177	95%
2002	302	233	77%
2003	729	679	93%
2004	1,065	923	87%
2005	1,493	648	43%
2006	1,304	547	42%
2007	1,220	496	41%
2008	908	370	41%
2009	836	360	43%
2010	1,146	610	53%

**Table 3:** Top 20 countries in the ISRCTN

Country	2000	2001	2002	2003	2004	2005	2006	2007	2008	2009	2010
UK	145	183	228	677	923	675	552	507	372	378	608
Netherlands			3	4	17	356	348	239	33	37	50
Germany			3	15	15	76	73	97	92	83	68
Canada		1	9	6	16	138	45	54	55	29	27
Switzerland			2	3	28	45	44	39	33	27	35
USA			9	10	16	50	44	20	32	24	27
Spain					3	13	23	19	26	30	40
Australia		1	4	8	13	36	13	17	12	8	17
Italy			3	1	6	9	9	18	18	29	29
France			1	1	1	8	18	18	29	16	25
Sweden				1	1	4	12	22	30	23	21
Belgium			4		2	8	9	10	22	13	15
Brazil			1		1	2	11	9	20	8	15
China						3	8	15	14	8	17
Finland			1		2	3	11	11	9	10	13
Denmark			2		3	4	7	10	4	7	17
India			1		1	9	9	14	12	1	9
Ireland						1	1	6	12	14	16
Norway			1	1		4	7	15	8	1	3
S Korea								6	10	8	10
Others	0	2	30	2	17	49	60	74	65	82	84
Total	145	187	302	729	1065	1493	1304	1220	908	836	1146

**Table 4:** Numbers of interventional trials and observational studies in ISRCTN

Year	Intervention trials	Observational studies
2000	145	0
2001	187	0
2002	302	0
2003	727	2
2004	1062	3
2005	1491	2
2006	1275	29
2007	1175	45
2008	861	47
2009	791	45
2010	1037	109

**Table 5:** Numbers of trials registered prospectively and retrospectively in the ISRCTN

Year	Prospective	Retrospective
2000	21	124
2001	37	150
2002	47	255
2003	51	678
2004	99	966
2005	247	1,246
2006	385	919
2007	450	770
2008	453	455
2009	440	396
2010	433	713

## Trial registration in the United Kingdom and overall challenges

There is no legal requirement to publicly register clinical trials in the UK. However, a number of organizations will collect different sets of information and any clinical researcher must follow strict procedures for the ethical review of their trial protocol (10). The Chief Investigator must apply for ethics approval. This should be made via the Integrated Research Application System (IRAS) which provides a ‘one-stop shop’ showing all the relevant permissions and approvals needed, including the clinical trial authorization to be submitted to the Medicines and Healthcare products Regulatory Authority (MHRA) (11). Research studies are reviewed by NHS Research Ethics Committees (RECs) established under policy from the relevant Health Departments in each of the four UK nations. The National Research Ethics Service (NRES) is the administrative body responsible for providing advice, assistance and operational support to NHS RECs for the whole of the UK (12).

The ISRCTN register is in regular contact with both the IRAS and the NRES organizations to ensure that systems whose aim is to facilitate the running of clinical trials in the UK also promote public registration and include trial IDs in their datasets and to discuss the feasibility of regular data exchanges.

Increased participation in trials is very much supported by the UK government (13). In 2010, the decision was taken to develop a UK Clinical Trials Gateway (UKCTG) aiming to provide a list of the trials that have enrolled or are enrolling UK participants, in a single environment (14). The sources used are the UK-based international ISRCTN register and the US-based ClinicalTrials.gov register (15). CCT provided data and consulting services to the Department of Health to ensure the launch of the UKCTG website in March 2011. The existence of the UKCTG has been noted in the Government's Budget for 2011 (16).

All European trial regulatory agencies contribute to the European EudraCT database. In March 2011, the European Medicines Agency (EMA) launched a public view of this data as the EU-Clinical Trials Register (17).

Some of the challenges that remain include:

- Continue to promote prospective registration. The focus should be to promote registration with health research funding bodies. There have been marketing efforts in the past and a lot will depend on the overall position of those organizations regarding mandating free access to ongoing and published research.
- Build on the increased availability of information on pharmaceutical industry trials through the recently launched European Register.
- Manage expectations in terms of completeness and quality of data, which is a resource intensive activity for registers (18).
- Encourage trialists to record complete and high quality data in trial registration databases, such as methodologic information, so that trials can be critically appraised (19).
- Ensure that more potential participants are made aware of research that is relevant to them by making sure that clinical trial information is presented in lay-friendly terms, an aspect considered key to the success of the UKCTG. Acknowledging that the scientific format of trial information might not facilitate increased participation, the ISRCTN register has also been working on introducing a new field to allow researchers to provide a lay summary of their trial.
- Strengthen the link between a registered trial and its resulting publications. Trial registration databases in partnership with journals have an important role to play in increasing transparency in trial reporting and reducing reporting bias (for a review see (20)), to improve the medical evidence base and, ultimately, patient care. By improving links between trial registers and resulting publications, readers and systematic reviewers of the medical evidence will have better access to information that is used to inform practice decisions. Electronic journals with unlimited space also have the opportunity to report all scientifically sound research, regardless of the outcome of the trial. BioMed Central has put into practice one of the original aims of the ISRCTN scheme by quoting trial IDs on an increasing number of articles (over 200 papers published quoting the ISRCTN trial ID, over 70 with the ClinicalTrials.gov trial ID). BioMed Central is also taking this further and recently launched its threaded publications concept (21), in which the ISRCTN register provides the first step in a sequence of ‘threaded’ electronic publications (22) relating to a particular trial of a particular treatment. Any publications – protocols, results, secondary analysis, case reports and datasets – that include the ISRCTN Trial ID in the abstract are hyperlinked back to the trial database, and the same system operates for the three other largest global trial databases, including ClinicalTrials.gov. BioMed Central has also been promoting and providing guidelines for the principle of raw clinical data sharing and publication, which represents maximum transparency in clinical research, as data are available for alternative analyses, external scrutiny and for the simplification and enhancement of systematic reviews and meta-analyses (23). This focus has helped facilitate the publication, as a stand-alone peer-reviewed article in the journal *Trials*, the individual participant data (more than 19,000 patients) from one of the largest trials ever conducted in acute stroke (24). As clinical data sharing and publication becomes more common and with effective links between journal publications and trial registration databases, there are real opportunities to enhance the medical literature.

## Conclusion

Although there are many expectations for the number, quality and accessibility of clinical trials, the volume of information that is publicly available has evolved dramatically over the past decade. Progress has been made in defining and applying standards. Science publishers have a role to play in requesting evidence of prospective registration and in facilitating the sharing and publication of all elements of trials, from study registration through to the raw data.

## Competing Interests

The ISRCTN Register is administered by Current Controlled Trials. Both CCT and BioMed Central have been part of the Springer Science+Business Media since 2008. HF and IH both employees of BioMed Central.
